# Behavioral Assessment and Evaluation of Innovative Hollow Glue-Laminated Timber Elements

**DOI:** 10.3390/ma14226911

**Published:** 2021-11-16

**Authors:** Nikola Perković, Vlatka Rajčić, Monika Pranjić

**Affiliations:** Structural Department, Faculty of Civil Engineering, University of Zagreb, 10000 Zagreb, Croatia; vlatka.rajcic@grad.unizg.hr (V.R.); monika.pranjic@gmail.com (M.P.)

**Keywords:** timber, modular, innovative, hollow, glue-laminated timber, FEM, climate change, carbon

## Abstract

Due to the growing need to preserve our planet and reduce carbon emissions during construction, the use of the only carbon-absorbing material, timber, is increasingly being imposed. In addition to the requirement of reducing emissions, there is a necessity for the shortest possible construction time and the minimum use of construction machinery, which has led to the development of prefabricated construction systems. This paper deals with the innovative, hollow, glue-laminated timber elements which are intended for modular construction. Comparing this new system with existing modular systems, the main features and behavior of the constitutive elements, i.e., the hollow, glue-laminated timber elements, are presented. Experimental and numerical analysis of the mechanical performance of the timber elements was carried out and a comparative analysis of the behavior of two different types of hollow timber elements was conducted. The finite element method was used to predict the behavior of this innovative structural system. The results are compared with the analytical procedure to provide a background for the development of standardized methods for the design of timber structures.

## 1. Introduction

**Figure 1:**
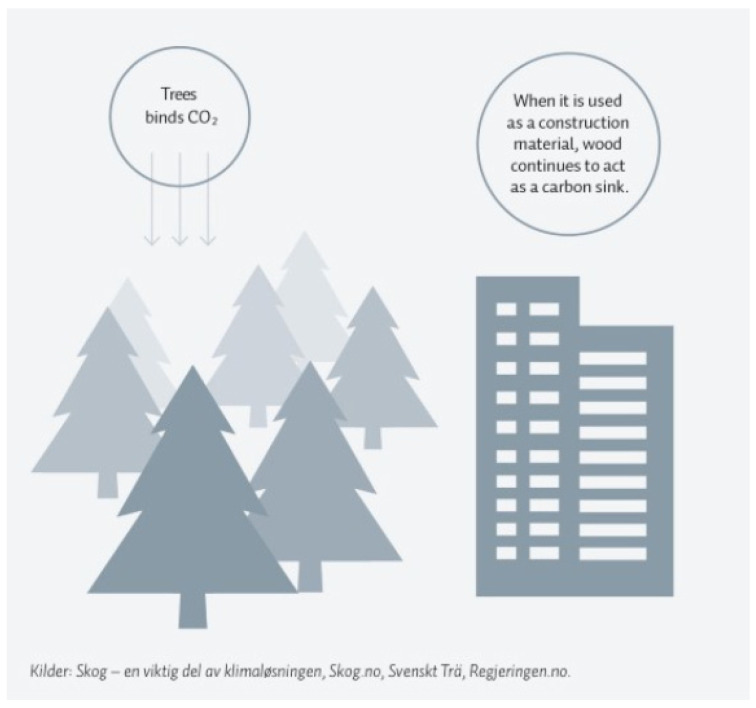
The impact of forestry on the climate [2].

Over the last decade, the number of massive timber buildings designed and constructed using cross-laminated timber (CLT) and glue-laminated timber (GLT) has increased significantly. The main advantages can be seen in low-rise timber construction, which could have a great share of the carbon sequestered in the global fight against climate change and global warming [1]. Carbon storage in the forest occurs through the accumulation of biomass in trees during photosynthesis. At the end of the cycle, wood releases stored carbon into the atmosphere through decomposition or combustion. Using wood in the construction of structures (and recycling it into furniture, panels, or other products after the building is demolished) can maximize the carbon storage effect (Figure 1) [2]. The amount of carbon dioxide emitted would be reduced by 25% if only 10% of new houses were built with wood [3]. Trees absorb the most carbon dioxide as they grow. Therefore, by planting two new trunks for each felled one, the absorption of carbon dioxide would be more than twice as high [2].

In addition to the impact on carbon dioxide emissions, it is important to consider the amount of energy required to process the building material. The tree leads here again. From 30% to 40% of the trunk itself is used for timber elements, and the rest is not thrown away. Rather, it is used to produce heating pallets, while sawdust can be used as an excellent insulating material.

**Figure 2:**
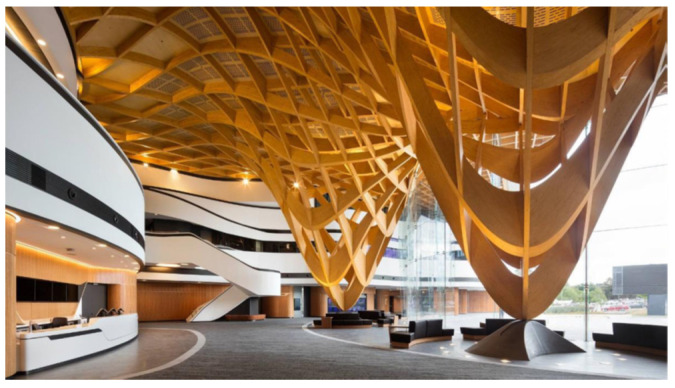
Bunjil Place, Melbourne.

Furthermore, timber structures may satisfy architectural aspirations, and may ultimately result in reduced costs and higher construction speeds, compared to standard, more common forms of construction. To make the most of the benefits of timber, in addition to sawn timber, wood-based products, in the form of different versions of glue-laminated timber, have also been developed. They open up the possibility of various shapes and options for cross-sections depending on one’s needs, almost without restrictions. It is possible to use curved elements that significantly improve the aesthetic impression [4]. An example of one such aesthetically interesting building is given in Figure 2.

**Figure 3:**
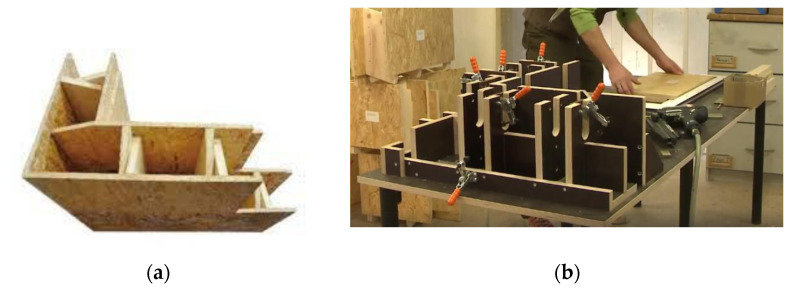
LUXHOME [7]. (**a**) corner element and (**b**) product assembly.

Another important advantage of wood is the extremely favorable ratio of its weight and strength. This advantage opens the possibility of building timber structures on soils of poorer quality or on larger slopes, where it would be much more complicated to create a classic concrete structure. Wood is a material that possesses two additional, significant abilities. One is the ability to exchange air with the environment and filter it through the building, and the other is the ability to control the humidity in the air. Thus, wood as a material creates a very pleasant atmosphere for life, which increases significantly with the number of exposed surfaces inside the structure which are treated with natural materials [5]. In addition, wood provides excellent thermal and sound insulation and does not emit harmful radiation [4]. Apart from its small weight, the advantage of timber is its elasticity, which makes it favorable compared to other materials during earthquakes [6]. As far as fire resistance is concerned, wood does not significantly change its mechanical properties while burning. The reason for this is that, shortly after the fire, a carbon layer is formed on the surface of the wood which prevents the development of heat in the interior [6]. Although timber possesses all of the above advantages, it has been neglected and avoided as a building material. Marija Kitek Kuzman surveyed the public’s attitude towards timber construction, asking about six hundred respondents in Slovenia. This study concluded that one-third of the respondents would decide to build with wood, while the rest would choose other (standard) types of construction. The main reason for this is ignorance of the properties of wood as a material, which was confirmed by the survey, because slightly less than half of the respondents were not familiar with its properties. Their greatest concerns were earthquake resistance, fire resistance, and the fear of expensive and frequent maintenance due to various pests [3]. Currently, there are several available innovative wooden elements for modular houses. “LUXHOME” has developed a modular system for building wooden houses from “wooden bricks” (Figure 3a) [7]. The production itself is automated so that by defining the dimensions of individual parts, the quantities of materials and costs are obtained immediately (Figure 3b), which can significantly reduce the amount of waste material. In addition to making wooden bricks, they also make floor and ceiling elements, windows, doors, insulation, and other similar products. 

**Figure 4:**
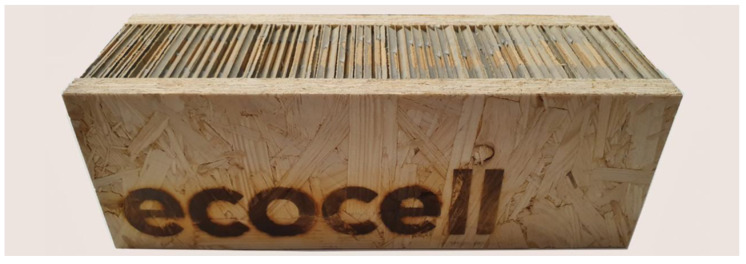
ECOCELL [8].

The next system is called “ECOCELL” [8]. This is an SIP system (structural insulated panels) which consists of an insulating material that is placed between two boards, most often OSBs (Figure 4). They are manufactured under controlled conditions and can be suitable in almost any construction. The system is solid, energy efficient, and very affordable. Most SIP systems use oil-based foam insulation, while “ECOCELL” has developed an innovative insulation system, the first that is non-oil based. Insulation makes up 75% of the volume of the element. The insulating matieral is in the shape of a honeycomb and is made of 100% recycled paper, in the form of corrugated cardboard, which is covered with a mineral coating (Figure 4).

**Figure 5:**
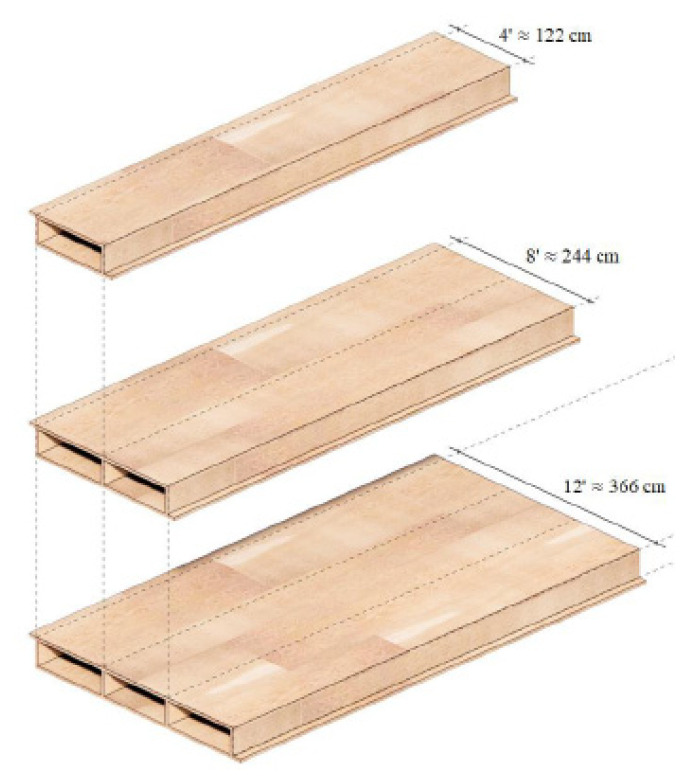
Wooden box elements [9].

Fabric Workshop is a company that designs its system of hollow-core, solid wood panels (Figure 5), columns, and walls, for which 50% fewer wood fibers are used, and the total cost is 10–35% lower compared to CLT [9]. Due to the cavities and box shape, this system has a better strength/weight ratio than comparable steel and concrete systems. The elements are pre-manufactured, so there is no use of saws and hammers on the construction site. Rather, these large boards are assembled and connected with metal fasteners. Although the material is expensive, there are significant savings in execution speed and error prevention due to this design method.

**Figure 6:**
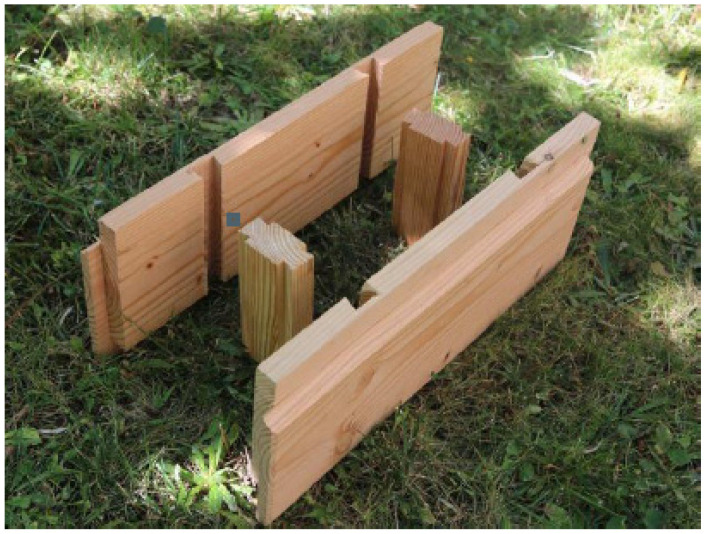
Wooden box elements [10].

“BRIKAWOOD” [10] is a wooden brick building system, developed by the French company Catharhome, which allows you to quickly build a house without the use of nails, screws, or glue. Each unit consists of four parts: two side elements and two transverse spacers that fit into each other with the help of a classic dovetail joint (Figure 6).

**Figure 7:**
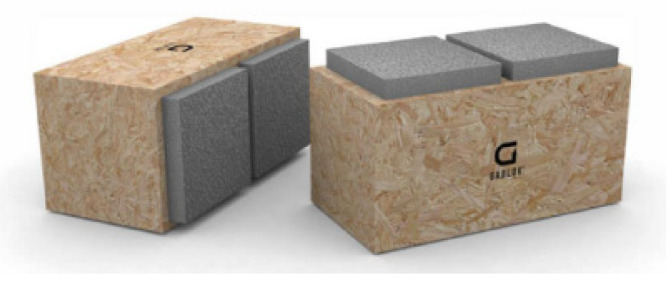
Gablok wooden box elements [11].

Another interesting system is “GABLOK” (Figure 7), which are insulated wooden blocks, designed in Belgium from recyclable materials [11]. Gablok has made the choice of using expanded polystyrene with a graphite additive (EPS) for its building system. The basic product has the form of styrene beads. Gabloks are available in three different lengths: 30 cm, 60 cm, and 90 cm. The height and width of individual blocks are always the same, 30 cm. An insulated wooden block of 60 cm weighs only 7.5 kg, which makes it very easy to handle, with no cranes needed on the construction site. The combination of wood and EPS provides significant insulation value, which can be further increased by the use of insulating materials on the outside of the blocks.

**Figure 8:**
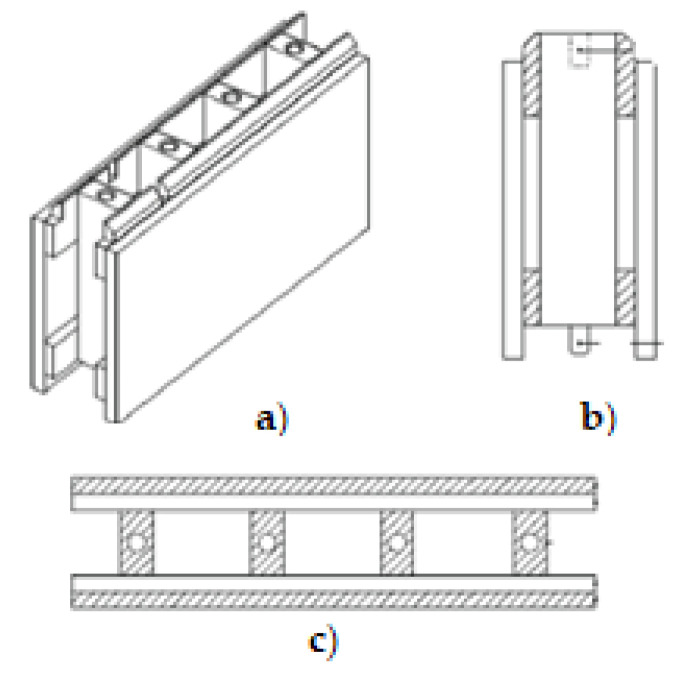
STEKO^®^ wooden box elements [12]. (**a**) Isometric view, (**b**) front view and (**c**) top view.

Finally, STEKO^®^ is a modular building system developed in Switzerland [12]. The external and internal walls are built of standardized, industrially produced modules, i.e., sustainable plantation wood from the Baltic. They are easy to install, even without the use of glue, nails, or similar fasteners. They consist of five parts that are glued together with non-toxic glue (Figure 8). Part one is made of C24 wood, the other parts are made of C16 wood, while the dowels are made of poplar. STEKO^®^ modular building system is suitable for residential buildings, as well as for communal, agricultural, industrial, or business buildings up to 10 storeys high. Furthermore, wooden modules can also be used for temporary buildings, fair and stage buildings, renovations, outbuildings, room partitions, or as a filler for skeletal structures.

The main reason for writing this paper is to show how hollow, glue-laminated timber elements are significantly different from others available in the world. Experimental tests are presented in order to develop manual calculations and to calibrate FEM modelling methods, helping to achieve the optimization of the methods, which can be adapted depending on the required function of the element. The dimensions of individual lamellae, and the materials from which they are made, can significantly affect the stress and displacement values, so the shape of the cross-section could be adjusted depending on the required characteristics, as described below.

When it comes to experimental investigation, many researchers have conducted similar tests, but it is difficult to find literature related to the perforation of timber elements. Fonseca et.al. [13] analyzed the simply supported wooden beam at ambient and high temperatures. In accordance with EC5 and elastic and plastic beam theory, the results, when compared, showed that the accuracy is satisfactory until the moment when instability is lost. In addition, the strength of the timber, along with the strength of the cross-section, decreases with an elevation of the temperature. The results show that experimental research is conservative, so this is a safer way to design.

Wan Mohamad et al. [14] showed that there are no major differences in strength between the Malaysian hardwood resak and keruing timber beams that they tested, although, theoretically, they are grouped differently. However, regarding the serviceability limit state, the Malaysian hardwood resak showed better performance. An important conclusion is that the failure occurred in the timber, not the adhesive, which indicates good and correct production of the elements.

A great contribution to the investigation into the strength of glue-laminated timber beams with round holes was made by Okamoto et al. [15]. Differences between homogeneous and heterogeneous timber grades were explored. The load-bearing capacity ratio between the homogeneous and heterogeneous timber grades ranges from 0.87 to 1.08. When it comes to the material and strength of the inner lamellae, where the modulus of elasticity in both types of specimen is equal, the ratio is between 1.13 and 1.21.

Finally, the tensile strength perpendicular to the grain is lower in heterogeneous timber grades. FEA analysis was also performed, where the above mentioned ratio is a little smaller in relation to the result given by the Bernoulli–Euler theory, because the hole size increased.

Efforts have been being made to model various problems in order to save the significant amounts of money needed to conduct experimental tests. In order to make the model results as accurate and credible as possible, computer FEM (finite element method) programs are used, i.e., computer programs based on the finite element method. 

### 1.1. Abaqus

Abaqus is a finite element analysis software whose main capabilities are the inclusion of linear and nonlinear analyses, large deformations and contact simulations, the availability of different types of analyses, graphical interface adjustments, and a wide range of nonlinear material possibilities. Accurate, robust, high-performance solutions for challenging nonlinear problems, large-scale linear dynamics applications, and routine design simulations may be delivered with the software [16]. In addition to the selection of materials from the library, the detailed creation of materials using several variables is allowed by Abaqus. For simple analysis, the material can be defined as linear elastic, while for more complex ones, it can be defined using a function calculated by the program itself or be defined as a function of an independent variable, such as temperature, but many more options are also available [17].

This software was also used in the research of Jordaan, JP [18], due to the complexity of the geometry, for modeling a beamthat was bent in four points in order to test the fatigue resistance. As the pattern is biaxially symmetrical, only half is modeled in Abaqus, while the finite element mesh is quadratic hexahedral in shape. On contact surfaces, exponential pressure is defined, and tangential behavior without friction is examined. The difference between the measured and calculated deformations (and thus the stresses) is about 7%.

Khorsandnia, Valipour, and Crews [19] modeled the composite timber–concrete beam using the finite element method and subsequently performed a manual calculation according to Eurocode 5 [20] to compare the results. The wood was initially modeled so that the modulus of elasticity, displacement, and Poisson’s ratio had different values for the three main directions (longitudinal, tangential, and radial). It was subsequently concluded that the mechanical properties of wood, in the direction perpendicular to the grain, have little effect on the beam response globally, and the isotropic characteristics of the material for beams without knots and defects and with dominant bending provide an error of less than 5% compared to orthotropic beams. Most of the mechanical properties of timber are taken from the test results. The concrete and timber parts were modeled as two separate bodies, and in the places of mechanical joints, springs that prevented only vertical movement were modeled.

The possibility of fracture modeling by bending softwood using the ABAQUS FEM code was also investigated [21]. Hill’s function is applied to describe the anisotropic plastic behavior of the material after the yield strength has been reached. As this function does not distinguish between compressive and tensile strength, the pressure and tensile zones are theoretically separated. For the needs of the model, the modulus of elasticity along the grains was determined, because it has the greatest influence on deformations, even in the orthotropic model, and on the modulus of rupture, i.e., the flexural strength. The numerical model consists of a beam bent at three points, where no displacement is allowed at the points of support, while a coefficient of friction is defined at the point of force input. A finite element mesh consists of elements with 20 nodes and 27 integration points (C3D20). Four such elements are located at the height of the sample, thus obtaining results identical to the analytical ones.

### 1.2. Ansys

Many researchers found Ansys software to be the most appropriate for modeling different materials and elements. In order to find the optimal solution for the application of sandwich panels as a trailer cover, a study [22] was conducted in which different types of panels were experimentally tested. After that, a numerical model was made in Ansys, for which data were obtained through tension tests. The 2D model simulated the four-point bending, and the results were compared with tests. The laminate material is defined as linear orthotropic, while the core material is linear isotropic. One support has fixed displacements in all directions, while the other has only vertical displacements fixed. The finite element mesh size is 1 mm. Obtained results were used for the optimal design of sandwich panels. A 3D model was made to test the panels in real conditions.

Bastola, Souliman, and Mohamed [23] modeled a four-point bending beam in Ansys to assess the fatigue behavior of asphalt material. Contacts between bodies are defined in such a way that neither displacements nor sliding of one element on another is allowed. The forces were set as constant, while the boundary conditions simulated a simply supported beam. 

Bano, Arriaga, Soilan, and Guaita [24] analyzed different models of timber beams with nodes using the finite element method to predict the failure load and crack location as they related to the size and position of the nodes and grain deflection in the node area. The orthotropic and elastoplastic behavior of wood was modeled using the Ansys parametric design language (APDL) command. The geometry is defined according to EN 408 for four-point bending with steel plates modeled at the support points to avoid local stress concentration, while the load is entered as pressure along the line, or as a concentrated load in thirds of the span. The finite element grid is quadrangular in shape so that it is as uniform as possible. The load is set to increase at each step until the utilization is 100%. The results of the numerical models were compared with those obtained by the tests, resulting in a difference of less than 9.7%.

Using Ansys, research [25] was conducted in which the bending capacity of timber beams with initial imperfections was examined. The beams have been used for about eighty years, having been taken from an old library, and their mechanical properties have been tested using non-destructive methods. They were subsequently introduced into the numerical model when timber was defined as an anisotropic material with elastoplastic behavior. According to the results of these non-destructive methods, the beams were divided into zones with different strengths and as such modeled. If part of the cross-section of the beam has collapsed or there were cracks or knots, they were modeled as such. The obtained values of the numerical model are 22% higher than those obtained by tests.

## 2. Innovative, Hollow, Glue-Laminated Timber Elements

According to the previous section, innovative systems for the construction of modular timber houses are increasingly being developed around the world. The goal of most manufacturers is to develop elements that will simplify construction, i.e., achieve the highest possible construction speed for the lowest possible cost. Guided by similar principles, an innovative, hollow, glue-laminated wood element has been developed. 

The novelity and the scientific contributiuon is reflected as follows:

- The innovative nature of the timber elements, which represent a new generation of timber products that enable easier construction, assembly, material savings, and global environmental sustainability.
- Experimental tests of hollow timber elements were performed to investigate the different possibilities of the type and arrangement of cavities that affect the load-bearing capacity and serviceability of the innovative hollow elements.
- Development of a numerical model for the purpose of simulating the behavior of hollow timber elements. The simulation results are compared with the experimental results and the model will be calibrated as needed so that a more extensive parametric analysis can be performed.
- Dimensions of individual lamellae and the material from which they are made can significantly affect the stress and displacement values, so the shape of the cross-section can be adjusted depending on the required characteristics.
- The possibility of achieving different cross-sectional characteristics, depending on the purpose of the element (please see discussion paragraph).

Cavities significantly reduce the weight of an individual element making it transferable by one or two persons, which eliminates the need for cranes, and also makes construction cheaper. The weight per meter for such hollow elements is approximately 6.5 kg. Table 1 shows the density values obtained by measuring the dimensions and mass of the non-perforated samples. 

**Figure 9:**
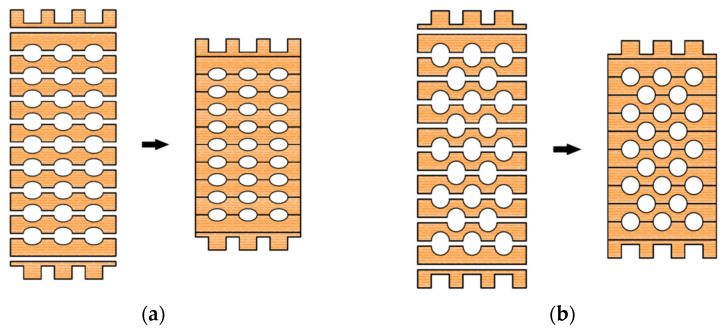
Glue-laminated hollow timber elements: (**a**) elliptical cavities; (**b**) circular cavities.

Elements consist of 20 mm-thick lamellae in which elliptical or circular cavities are incised (Figure 9). In each lamella, the grains are directed along its length and are interconnected by gluing. The lamellae are obtained from recycled waste material, and the sawdust formed by the incision of the cavities can be used as an insulating material or be processed into pellets.

The first and last lamellae are shaped so that, when stacked on top of each other, the elements fit together. In this way, fast and efficient construction is enabled without the use of fasteners. In the other lamellae, semi-elliptical or semi-circular shapes are cut and then glued together to obtain the desired cross-section with elliptical or circular cavities.

As the samples with elliptical cavities generally behaved better during experimental tests, the accuracy of the assumptions embedded in the numerical calculations—that the elements function as mechanically assembled beams, based on the standard EN 1995-1-1—was studied in the example of one, as was numerical modeling. For elements with circular cavities, the analogy is the same, only the expressions for the circle are used in the formulas instead of the expressions for the ellipse.

### 2.1. Experimental Testing

#### 2.1.1. Strong-Axis Bending

**Figure 10:**
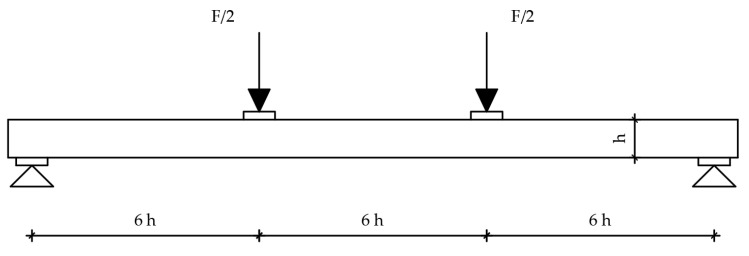
Four-point bending test setup.

A dynamic–static universal machine from Zwick Roell GmBH test & Co. KG, Germany was used for the experimental bending tests. The load capacity of the machine is 250 kN, class 1, which means it has a 1% deviation. This system enables the collection of force magnitude, displacement, acceleration, relative deformation, temperature, and stress values. The system is suitable for short-term and long-term static high-resolution measurements. The data collection system is MGC plus from the manufacturer HBM—Hottinger Baldwin Messtechnik, Germany. The load was defined by displacement control, and the speed was 6 mm/min. The load was applied over a steel beam, with an additional I profile, simulating four-point bending. A total of six measuring channels were used as follows: time, deflection on the supports and in the midspan, piston displacement, and load. Linear variable differential transformers with a precision of 10 mm (supports) and 100 mm (midspan) nominal values were used for deflection measurements. Since there are notches in the end lamellae of the samples, additional timber elements were made and placed at the load input area. The substitute elements are made of hardwood and geometrically correspond to the shape of the specimen, taking the form of notched connections. The surface of the element, which is on the side of the load cell, is completely flat. In this way, proper load input was enabled. Given the aim of this study, it was necessary to set up a system to prevent lateral-torsional buckling of the element at the required positions. Slender bending beams that have a large h/w ratio and which are loaded parallel to the weaker axis tend to have stability issues. This is due to the deflection of the compression chord. The beam has a lateral displacement with simultaneous rotation. Consequently, steel clamps are placed on the supports, covering a minimum of two-thirds of the sample height. Regarding the experiment’s consistency, load-midspan displacement responded as expected, and all beams presented similar stiffness up to a certain load level corresponding to the elastic response. For higher loads, their stiffness was considerably reduced due to local defects of the timber, cracking, and the adhesive behaviour. Failure occurred due to the pressure reaching the tensile strength in the lower zone of the specimen. To determine the mechanical properties of the wood, the four-point bending tests on the timber elements were conducted according to EN 408 [26] (Figure 10). The samples were simply supported with a span of 18 h, where h represents the height of the sample. Both the distance of the force from the support and the distance between the forces is 6 h. In the areas of force input, small steel plates were placed to prevent local stress concentrations, i.e., to achieve better force distribution, spreading it more evenly per sample. Maximum force was reached after (300 ± 120) seconds. Displacements were measured in the middle of the span and on the supports.

**Figure 11:**
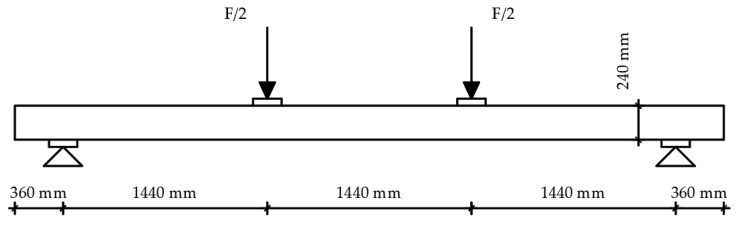
Strong-axis bending experimental test setup.

**Figure 12:**
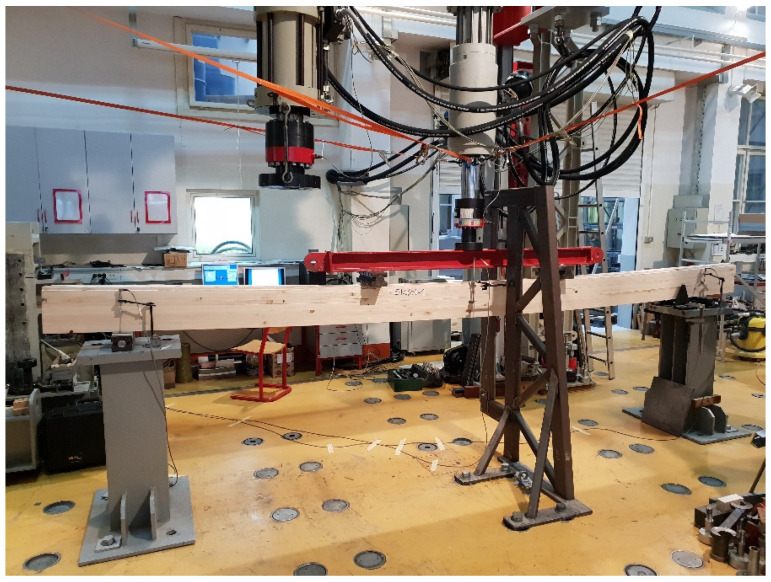
Timber beam during strong-axis bending experimental test.

For the strong-axis bending, the samples were laid in such a way that the larger cross-sectional dimension equals the height according to EN 408 [26], which is 240 mm. This determined the distance between the two concentrated forces and the distance of the forces from the supports. The width of the beam is 120 mm. All other dimensions are shown in Figure 11, while the sample during the strong-axis bending test is shown in Figure 12.

**Figure 13:**
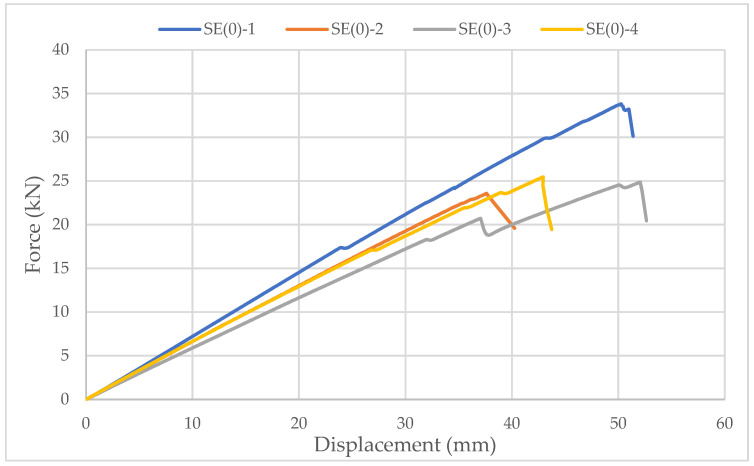
Force-displacement curve for strong-axis bending of samples with elliptical cavities.

**Figure 14:**
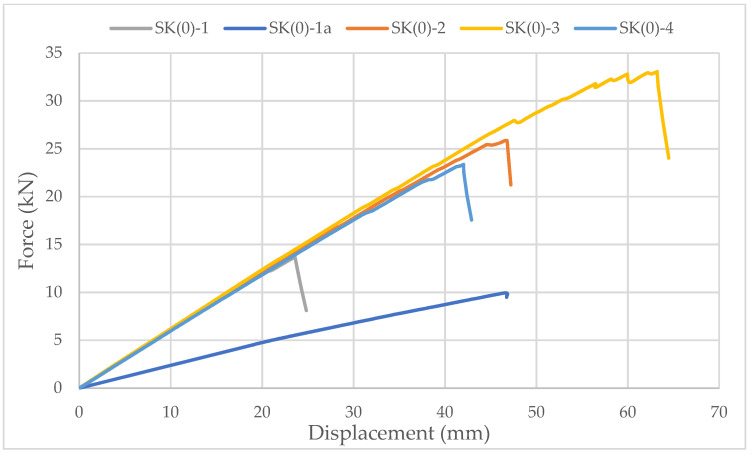
Force-displacement curve for strong-axis bending of samples with circular cavities.

**Figure 15:**
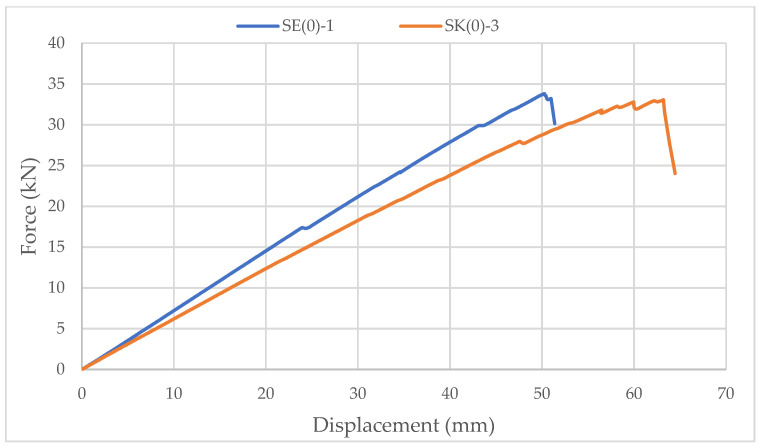
Comparison of the force-displacement curve for strong-axis bending of samples with elliptical and circular cavities.

Specimens with ellipse-shaped cavities, marked SE(0)-n, and circular cavities, marked SK(0)-n, were tested. The notation (0) represents strong-axis bending, while “n” represents the number of the tested sample. In Figure 13, Figure 14 and Figure 15, the results, according to the data obtained from the tests, are shown.

**Figure 16:**
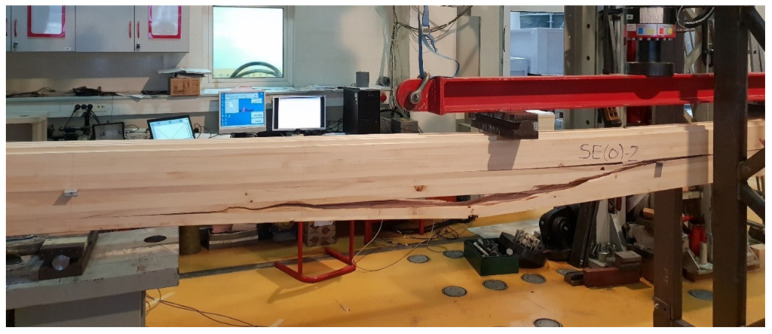
Failure of sample SE(0)-2 in tension.

The linearity of the force-displacement curve for the samples of both types of cavities indicates the ruling mechanism of failure due to the brittle behavior of the wood in the tension zone. Towards the end of the test, areas of force reduction at constant displacement are observed on the curve because of the failure of grains at the highest stress. At that moment, the cracking of timber appeared. The force then increases until the failure of the next grain, and so on, until the total failure of the sample occurs as it reaches its tensile strength (Figure 16).

The higher slope of the force-displacement curve for samples with elliptical cavities can be seen in Figure 15, which indicates their greater stiffness. The first reason is the perforation percentage of the samples with elliptical cavities. The second is the arrangement of elliptical cavities one below the other, which allows proper transmission of force along with the wooden web, which is not the case for samples with circular cavities.

The higher stiffness of samples with elliptical cavities can also be proved by the value of the force at which the maximum deflection (l/250) is achieved, determined according to the serviceability limit state. For the samples with elliptical cavities, the value of the force is 37% of the failure force, while for samples with circular cavities, it is 32% of the failure force. It should be noted that the stiffness of the perforated samples is approximately 30% lower compared to samples of the same dimensions without perforations.

In Figure 13 and Figure 14, it can be seen that the failure of samples with the same type of cavities does not occur at the same load value. The cause is mostly natural wood defects, such as knots, shakes, cross-grain, crookedness, rind galls, burr, and curl.

#### 2.1.2. Test of Compression Strength Perpendicular to the Grain

The Z600E Universal Static Testing Machine, Zwick Roell GmBH & Co. KG (Ulm, Germany) was used for this type of experimental test. The Z600E is a universal pressure-tensile testing machine with a capacity of 600 kN, and is electrically driven, designed for very precise static and low-cycle tests of smaller samples. A crosshead speed from 0.01 to 200 mm/min is possible. The maximum test frequency is 0.5 Hz and the accuracy of the set speed is 2.5%. The measured-value transmission rate to PC is 500 Hz. It has an upper test space for tensile tests (reinforcing steel and prestressing wood, plastic, composites, etc.) and a lower test space for pressure and bending tests of smaller samples. The machine is controlled by a PC using the “test expert” program. The program controls the force or displacement that is applied during the test protocol.

As it can be seen in Figure 18, as with the bending test, additional notched timber elements were made for this test to ensure the load input. The load speed was 1 mm/min, and the test lasted until the specimen failure occurred. The majority of the samples responded as expected, and the consistency can be seen in Figures 19 and 20, especially in the part of the branch with an elastic response, where all presented approximately constant stiffness up to the maximum load and similar displacements at failure.

**Figure 17:**
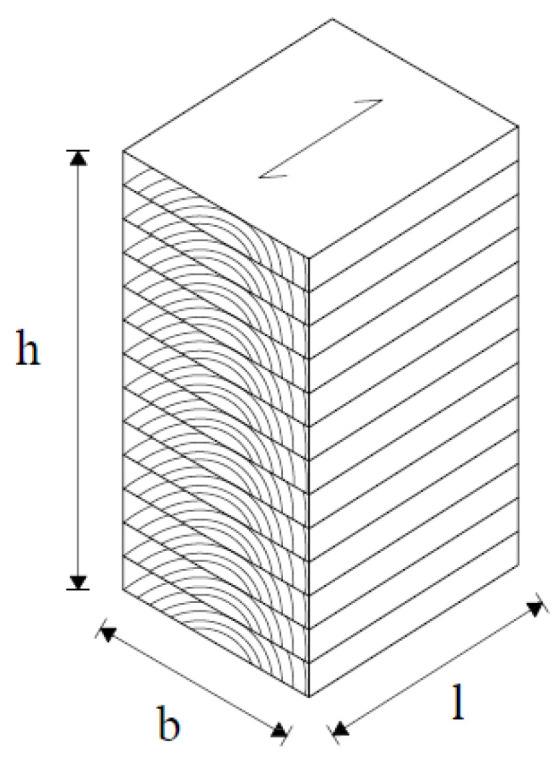
Sample for compression test.

Samples with elliptical and circular cavities have been tested in the direction of the strong and weak axis. The loading speed was set in such a way that the maximum force, i.e., the force at which the sample will fail, was reached in time (300 ± 120 s). The gauge length should be approximately 0.6 h, be located in the center of the test sample, and not be closer to the b/3 edges of the sample. An example of a laminated sample with marked dimensions is given in Figure 17.

#### 2.1.3. Direction of Weaker Axis

**Figure 18:**
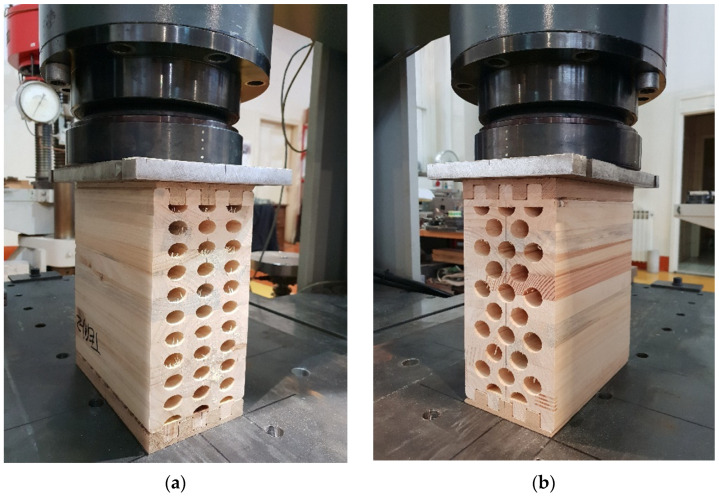
Timber samples during experimental test of compresion strength perpendicular to grain in the direction of weaker axis. (a) elliptical cavities; (b) circular cavities.

In this case, the sample is laid in such a way that the load acts in the direction of the weaker cross-sectional axis. The height of the sample (h) is 240 mm, the width (b) is 120 mm, and the length (l) is 210 mm. Figure 18a shows the specimen with elliptical cavities, and Figure 18b circular cavities.

**Figure 19:**
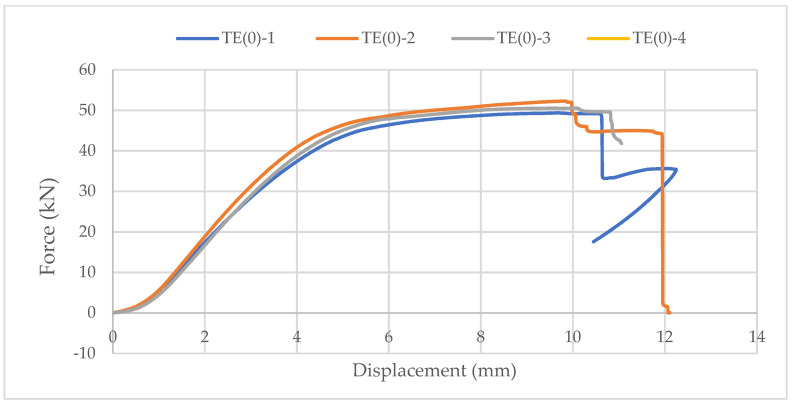
The force-displacement curve for compression perpendicular to the grain of samples with elliptical cavities in the direction of the weaker axis.

**Figure 20:**
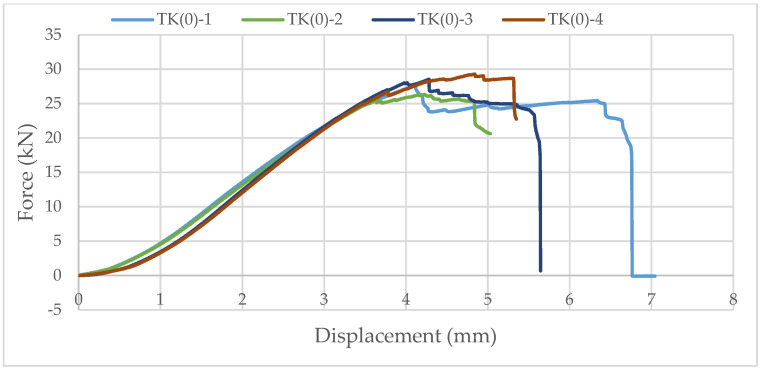
The force-displacement curve for compression perpendicular to the grain of samples with circular cavities in the direction of the weaker axis.

**Figure 21:**
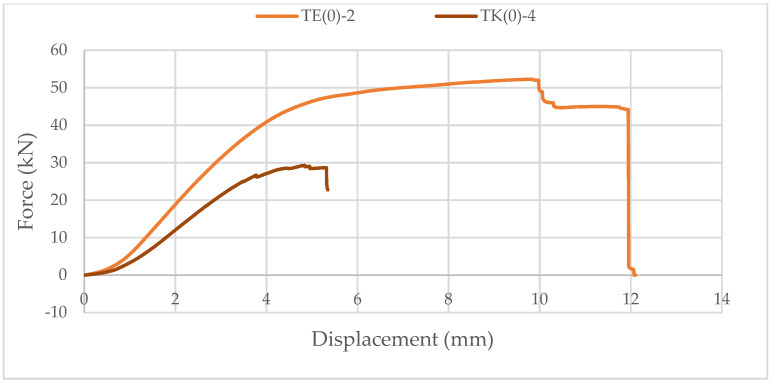
Comparison of the force-displacement curve for compression perpendicular to the grain of samples with elliptical and circular cavities in the direction of the weaker axis.

Samples with ellipse-shaped cavities, marked TE(0)-n, and circular cavities, marked TK(0)-n, were tested. The notation (0) represents compression in the direction of weaker axis, while “n” represents the number of the tested sample. In Figure 19, Figure 20 and Figure 21, the results, according to the data obtained by tests, are shown.

The force-displacement curve at the beginning shows adjustment and starts with a nonlinear diagram. Then, it shows linear behavior on the approximately 25% horizontal axes length. After that, the trend continues until end nonlinearity is detected, which indicates the ductile behavior of wood in compression. It can be seen in Figure 21 that the sample with elliptical cavities withstands twice the force compared to the sample with circular cavities because of the perforation percentage and the arrangement of the cavities, as explained for strong-axis bending.

**Figure 22:**
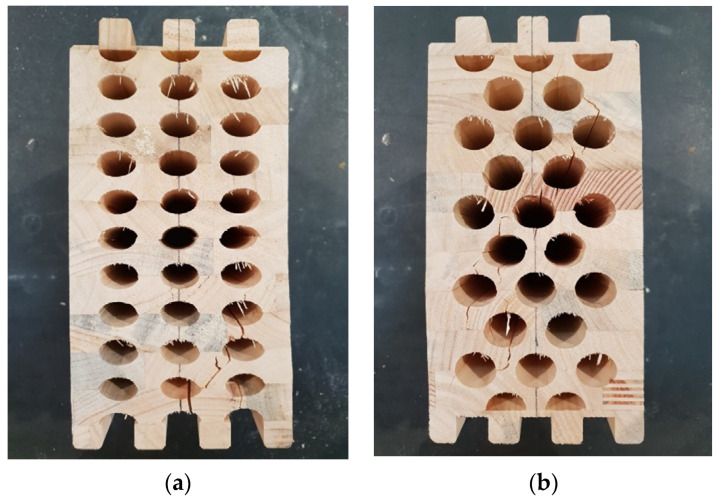
Samples with elliptical (**a**) and circular (**b**) cavities after experimental test of compressive strength perpendicular to the grain in the direction of a weaker axis.

In Figure 22, it can be seen that the cracks for the samples with elliptical cavities are almost vertical, while they are oblique for the samples with circular cavities, connecting the cavities and following an irregular path of force.

#### 2.1.4. The Direction of the Stronger Axis

**Figure 23:**
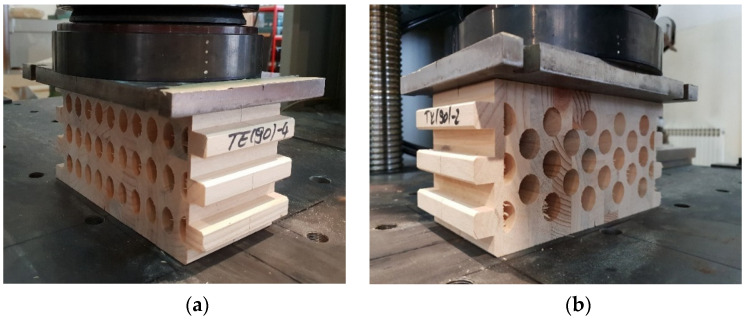
Timber samples during the experimental test of compression strength perpendicular to the grain in the direction of the stronger axis. (**a**) elliptical cavities; (**b**) circular cavities.

In this case, the specimen is laid in such a way that the load acts in the direction of the stronger cross-sectional axis. The height of the sample (h) is 120 mm, the width (b) is 240 mm, and the length (l) is 105 mm. Figure 23a shows the specimen with elliptical cavities, and Figure 23b circular cavities.

**Figure 24:**
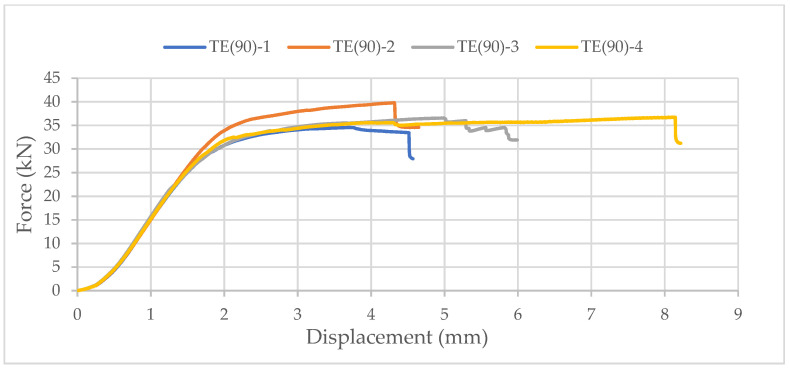
The force-displacement curve for compression perpendicular to the grain of samples with elliptical cavities in the direction of the stronger axis.

**Figure 25:**
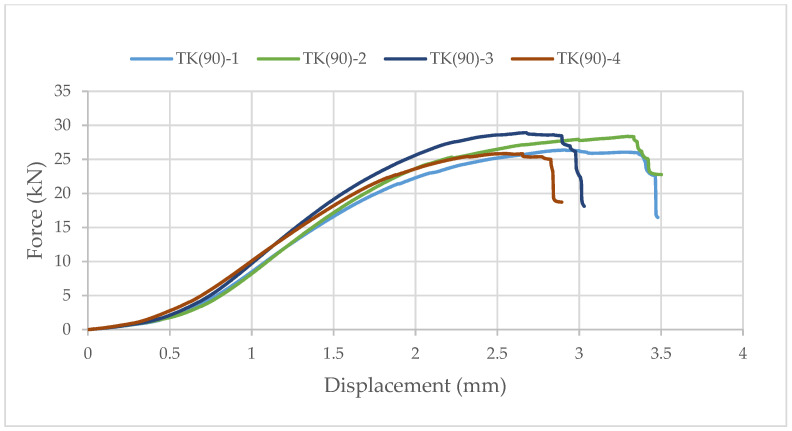
The force-displacement curve for compression perpendicular to the grain of samples with circular cavities in the direction of the stronger axis.

**Figure 26:**
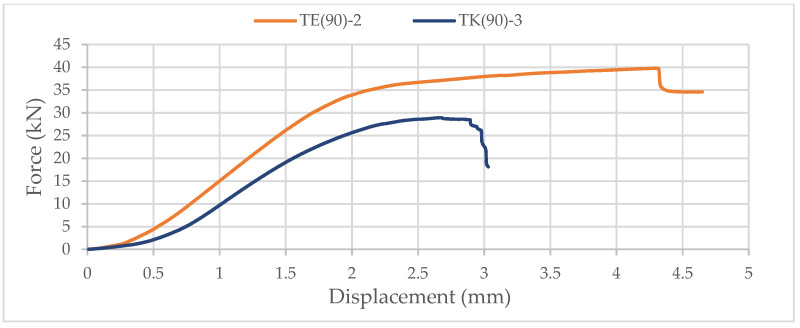
Comparison of the force-displacement curve for compression perpendicular to the grain of samples with elliptical and circular cavities in the direction of the stronger axis.

Again, samples with ellipse-shaped cavities, marked TE(90)-n, and circular cavities, marked TK(90)-n, were tested. The notation (90) represents compression in the direction of the stronger axis, while “n” represents the number of the tested sample. In Figure 24, Figure 25 and Figure 26, the results, according to the data obtained by tests, are shown.

The force-displacement curve has the same shape as in the previous case, but the difference in the applied load is not so significant. The reason for this is that the cavities are laid one below the other in both cases. Although specimens with circular cavities have a local increase in area in an area where there are only two cavities in cross-sectional height, the wooden web is narrower and slightly curved and the perforation percentage is larger so the force they can withstand is smaller.

**Figure 27:**
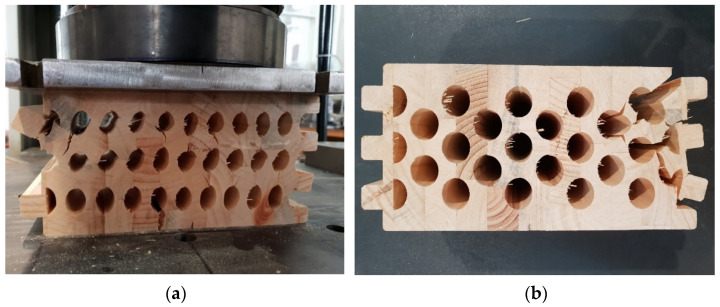
Samples with elliptical (**a**) and circular (**b**) cavities after experimental test od compressive strength perpendicular to the grain in the direction of the stronger axis.

The sample with elliptical cavities again has predominantly vertical cracks (Figure 27), while the cracks for specimens with circular cavities are oblique due to the slightly curved web, despite the position of the cavities one below the other.

## 3. Numerical Model of Innovative, Hollow, Glue-Laminated Timber Beam

The behavior of the samples with elliptical cavities was shown to be better, so an FEM model in Ansys was made only for that case. Out of all analysis types in Ansys, Static Structural has been chosen. It consists of six interconnected steps.

In the first step, “Engineering Data”, the material can be chosen or can be created manually, using data obtained from experimental tests. The timber is defined as an anisotropic material. The density was obtained by measuring the mass and volume of the samples, while the modulus of elasticity was calculated as a mean value from the force-displacement curve, according to the experimental test data.

**Figure 28:**
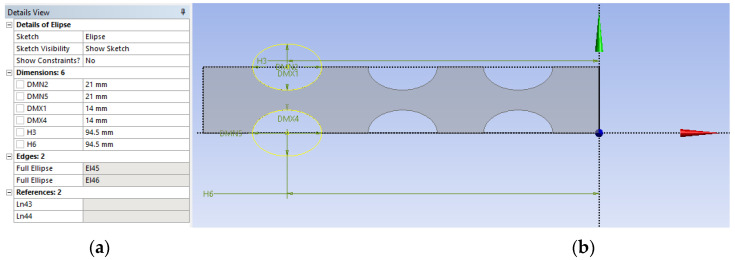
Parametric geometry definition in Design Modeler. (**a**) details view and (**b**) lamella geometry view.

The geometry is parametrically defined (Figure 28a) in the second step, using Design Modeler (Ansys 2020 R2, Canonsburg, PA, USA). It can thus be changed at any time so that the optimal shape and arrangement of the cavities can be found (Figure 28b).

In the next step, materials were attached to each of the created bodies. The connection between each lamella needs to be defined, according to the contact types shown in Table 2. It is generally assumed that the adhesive will not fail, but its characteristics need to be tested to determine its stiffness. Then, the behavior of the adhesive can be modeled using a frictional contact type.

**Figure 29:**
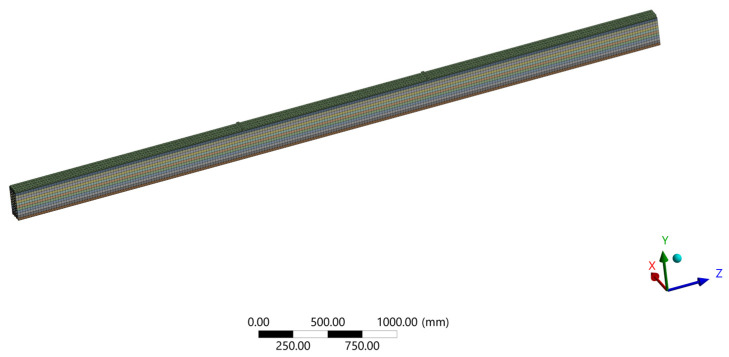
Mesh size.

Ansys provides general-purpose, high-performance, automated, intelligent meshing software that produces the most appropriate mesh for accurate, efficient metaphysical solutions, from easy, automatic meshing to highly crafted mesh. Smart defaults are built into the software to make meshing a painless and intuitive task, delivering the required resolution to capture solution gradients properly for dependable results [27]. Quadratic mesh with an element size of 20 mm has been chosen(Figure 29).

The load and boundary conditions were defined in the fourth step, ˝Model Setup˝. As the sample was simply supported, fixed support was selected at one end and displacement at the other, where only longitudinal displacement was enabled.

**Figure 30:**
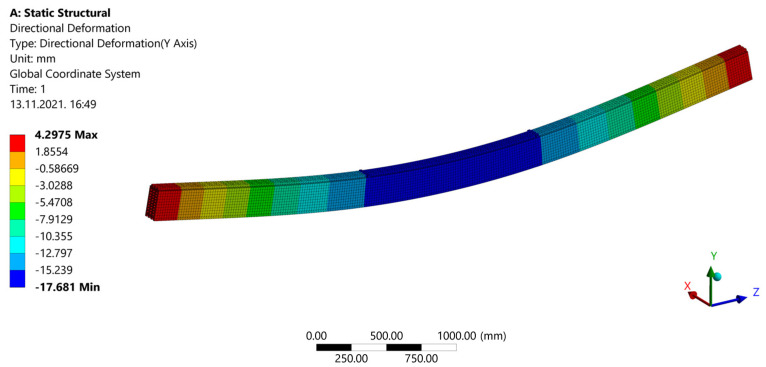
Deflection of the beam.

**Figure 31:**
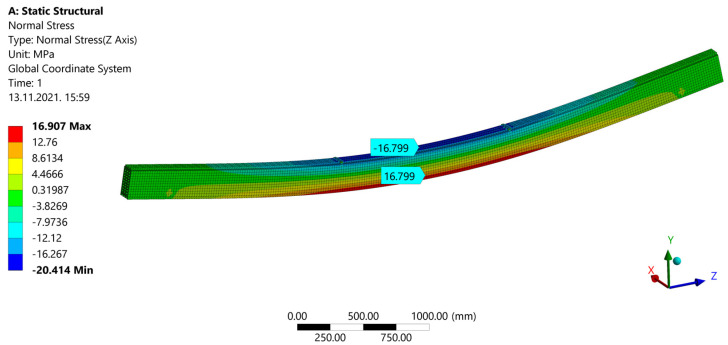
Normal stress.

**Figure 32:**
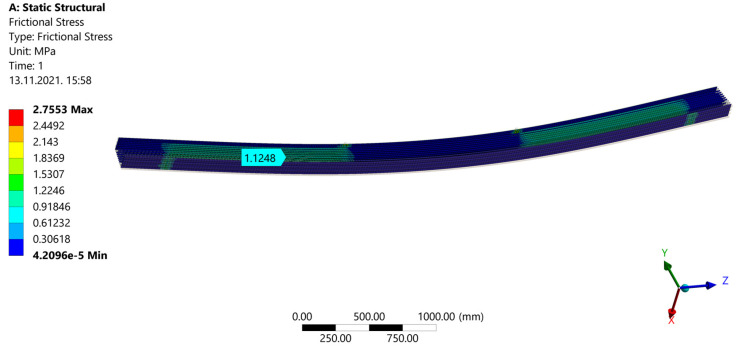
Shear Stress.

After a calculation during ˝Model Solution˝, results are shown. ˝Directional Deformation˝ can be seen in Figure 30. When ˝Normal Stress˝ is shown (Figure 31), areas of compression and tension can be observed. Compression areas are painted blue with a negative value, while red color and a positive value indicate tensile stress. In Figure 32, “Frictional (Shear) Stress” is shown.

Deformations obtained by the FEM model match very well with those from the experimental tests, with a deviation of 3.01% for the strong-axis bending (Table 3).

Stresses from the FEM model were compared with those calculated manually, using the procedure according to Annex B from EC5 for mechanically jointed beams. Maximum stress for the strong-axis bending deviates by only 1.24% (Table 4).

## 4. Discussion

The aim of the paper is not only to improve innovative elements but also to raise awareness of various issues through the prism of environmental protection and the rational use of natural resources. As there is a great untapped potential of energy production from renewable energy sources in the timber industry, the production of the elements is in accordance with European and world standards. The “waste” of the hollow timber elements is eventually used for the production of wood briquettes, in other words, of electricity and heat energy. Under high pressure, clean and dry sawdust is pressed and the briquette is made of natural timber and does not contain adhesives and similar substances, and as such has a wide application and can be used in all types of solid fuel stoves. In this way, the circular economy model has been adopted and thus the whole process was completed, in which there is no waste from the entry of the log to the exit of the finished product.

This paper provides data from experimental tests on innovative timber elements. Before preliminary tests, the density of the wood material was determined to confirm the timber class. After the delivery of the initial specimens, a four-point bending test was performed. Experimental research has shown that the beams with a circular profile geometry have approximately 60% of the load-bearing capacity compared to normal GL beams of the same cross-sectional dimensions, while beams with an elliptical profile geometry have approximately 70% of the load-bearing capacity compared to normal GL beams. During the test, it was noticed that the initial cracks were formed at the places where there were defects in the wood. Further tests were followed by testing the samples in compression perpendicular to the grains in the direction of the stronger and weaker axes. It can be concluded that a similar percentage of load-bearing capacity occurred in relation to normal GL beams, but only for beams with elliptical cavities, while beams with circular cavities showed significantly worse behavior, i.e., a load-bearing capacity of about 35% in comparison to normal GL beams.

When comparing the results obtained through experimental research and the model created in Ansys, very good matches were obtained. The mean values of the modulus of elasticity were experimentally obtained, and were then defined as such in the manual calculation and the numerical model in Ansys. When compared with the results of other girders tested in the laboratory, larger deviations would be obtained. For such a small number of samples, this is expected due to the characteristics of wood as a material, with all its defects and irregularities that may be present in it, and it would be necessary to take a much larger number of tested samples, for which the mean global values of mechanical characteristics would be selected for the calculation and modeling in Ansys.

During numerical modeling, certain stress concentrations were observed at the points of force applied to the girder. The reason for this is the input of the load via a very small geometry, i.e., via a single line. In reality, this is not the case, but various steel plates are installed in order to eradicate stress concentrations, and in this respect, it is possible to improve the model itself.

**Figure 33:**
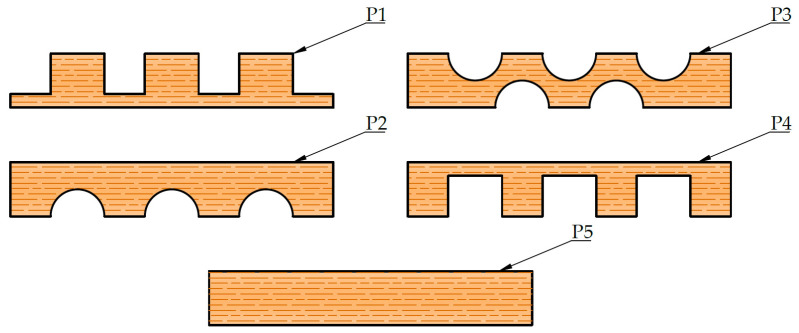
Lamella types.

This research provides a different approach due to the possibility of achieving different cross-sectional characteristics, depending on the purpose of the element (Table 5). If reduction of displacement is needed, the wood class of all lamellae may be increased. An increase in strength, with approximately the same deformation values, is achieved with higher timber classes of only the external lamellae. For reduction of both displacement and stress, the class of timber may be increased for inner lamellae. This could be achieved more efficiently by replacing the lamellae P3 with lamellae P5 or by increasing the thickness of the edge lamellae (see Figure 33). For drastic reduction of displacement and stresses, P5 lamellae, with greater thickness compared to others, should be added near the upper edge of the timber element.

In addition, perforated beams are longer than others available in the world, so it is possible to achieve faster construction. Finally, cavities can bring many benefits if used in the right way, such as the installation of insulation material, in order to increase the energy efficiency of the system.

## 5. Conclusions

Due to the growing pollution of the environment with carbon dioxide, the threat of global warming, and the desire for a healthier lifestyle, timber is the building material of the future. In addition, most people strive for savings in terms of materials, construction machinery expenses, but also the workforce itself, so the growing development of a modular way of building is expected. Therefore, timber is an ideal material from which to produce innovative elements for simple, fast, and efficient construction.

Each of the newly developed structure elements needs to be checked for mechanical properties using experimental tests. In most cases, several versions of the same system will appear, from which the most favorable one will be selected. Thus, in this case, tests have shown that specimens with elliptical cavities behave better than those with circular ones. In addition, it was shown that the adhesive used to make the samples, i.e., to glue the lamellae, was inadequate due to the occurrence of failures in the adhesive, which is unacceptable, so it was swapped during the development process for another type of adhesive which fulfills all requirements.

As the experimental tests are time-consuming and expensive, the aim is to develop new calculation methods and numerical models. In this paper, it can be concluded that hollow elements can be evaluated as composite girders because the matches with the results of experimental tests are strong. Numerical modeling using the finite element method in the Ansys software package also provided excellent matches with the test results, but certain corrections are possible when it comes to boundary conditions, in terms of reducing stress concentrations to obtain a better match with the results of calculations according to EN 1995.

In the future, a system of element shapes that are most suitable, depending on the position at which they will be used in construction, should be developed. Consideration should also be given to how a different size or arrangement of perforations affects cross-sectional properties. It is also necessary to test structural elements for lateral loads, such as a wall element. In addition, the behaviour of the elements in fire situations should be considered, as well as different fire protection systems. Details of corner joints and joints that connect vertical and horizontal elements should be analyzed. This would contribute to the reduction of the research gap that now exists, i.e., the lack of information on the testing and use of perforated wooden elements.

Finally, it should be emphasized that timber is a specific material. It is also anisotropic and heterogeneous, obtained from nature, which means that differences in the characteristics of the material are possible. In addition, there are numerous defects in the wood that weaken its properties. Therefore, it is necessary to pay great attention to the design of timber structures and all the variable parameters, but the material itself and all the benefits that life in wooden structures brings, are very much worth it.

## 6. Patents

The producer of the timber elements, a company (Tersa Ltd from Croatia), is in the application process for an intellectual property patent so that this product and system are protected.

## Figures and Tables

**Table 1:** Density of timber.

Specimen	Length (mm)	Width (mm)	Height (mm)	Volume (mm^3^)	Mass (g)	Density (kg/m^3^)
1	99.80	100.20	21.35	213,499.15	85.30	399.53
2	100.10	99.80	25.55	255,243.99	93.55	366.51
3	99.80	100.90	23.33	234,928.90	95.25	405.44
4	100.03	99.97	25.00	249,999.98	99.65	398.60
5	99.81	99.91	24.36	242,918.34	97.15	399.93
6	100.10	99.80	24.00	239,759.52	90.20	376.21
7	100.38	99.85	24.72	247,767.15	90.45	365.06
8	100.28	100.00	22.80	228,638.40	81.40	356.02
9	99.80	100.35	22.20	222,331.45	81.00	364.32
10	100.09	99.80	26.70	266,705.82	93.90	352.07
Average						378.37

**Table 2:** Types of contacts in Ansys.

Contact Type	Separation	Slip
Bonded	NO	NO
No separation	NO	YES (without resistance)
Frictionless	YES	YES (without resistance)
Frictional	YES	YES, with resistance if F_lip_ > F_friction_
Rough	YES	NO

**Table 3:** Comparison of beam deflection between experimental tests and Ansys.

Bending Axis	Deflection (mm)		Deviation (%)
	Experimental Tests	Ansys	
Strong axis	50.266	51.824	3.01

**Table 4:** Comparison of normal stresses between experimental tests and Ansys.

Position Edge/Connecting Plane	Normal Stress (N/mm^2^)		Deviation (%)
	Manual Calculation	Ansys	
upper edge	29.38	29.75	1.24
1	24.76	24.81	0.22
2	19.99	19.89	0.48
3	14.88	14.87	0.07
4	9.92	9.99	0.70
5	4.96	5.00	0.80
6	0.00	0.02	0.00
7	4.96	5.00	0.80
8	9.92	9.99	0.70
9	14.88	14.87	0.07
10	19.99	19.89	0.48
11	24.76	24.81	0.22
lower edge	29.38	29.75	1.24

**Table 5:** Possibilities of combining different timber classes and lamellae.

	Lamellae Height (mm)/Number/Wood Class					Girder Height (mm)	Girder Span (m)	Force (kN)	Displacement (mm)	Stress at the Edge (MPa)
Case	P1	P2	P3	P4	P5					
1	20/1/C20	20/2/C20	20/8/C20	20/1/C20	20/0/C20	240	4.5	14	48.3	25.2
2	20/1/C22	20/2/C22	20/8/C22	20/1/C22	20/0/C20	240	4.5	14	45.9	25.2
3	20/1/C35	20/2/C22	20/8/C22	20/1/C35	20/0/C20	240	4.5	14	41.4	29.6
4	20/1/C22	20/2/C35	20/8/C22	20/1/C22	20/0/C20	240	4.5	14	41.5	22.8
5	20/1/C22	20/2/C22	20/8/C35	20/1/C22	20/0/C20	240	4.5	14	42.1	23.2
6	20/1/C20	20/2/C20	20/6/C20	20/1/C20	20/2/C20	240	4.5	14	44.3	23.1
7	20/1/C22	20/2/C22	20/6/C22	20/1/C22	20/2/C22	240	4.5	14	42.1	23.1
8	20/1/C35	20/2/C22	20/6/C22	20/1/C35	20/2/C22	240	4.5	14	38.3	27.4
9	20/1/C22	20/2/C22	20/6/C22	20/1/C22	20/2/C35	240	4.5	14	37.9	20.9
10	20/1/C22	20/2/C22	20/6/C35	20/1/C22	20/2/C22	240	4.5	14	40.7	22.4
11	25/1/C20	20/2/C20	20/8/C20	25/1/C20	20/0/C20	250	4.5	14	41.6	22.5
12	20/1/C20	20/2/C20	20/8/C20	20/1/C20	20/2/C20	280	4.5	14	27.8	17.0
13	20/1/C20	20/2/C20	20/8/C20	20/1/C20	25/2/C20	290	4.5	14	24.7	15.6
14	20/1/C20	20/2/C20	20/8/C20	20/1/C20	25/2/C35	290	4.5	14	21.5	13.6
15	20/1/C20	20/2/C20	20/6/C20	20/1/C20	25/4/C35	300	4.5	14	17.3	11.3

## Data Availability

Data available on request due to restrictions, e.g., privacy or ethics. The data presented in this study are available on request from the corresponding author.

## References

[1] Buchanan A.H. Can timber buildings help reduce global CO_2_ emissions?. Proceedings of the 9th World Conference on Timber Engineering 2006 (WCTE 2006).

[2] On the Side of Nature. https://www.moelven.com/news/news-archive2/2019/on-the-side-of-nature.

[3] Kitek Kuzman M. (2010). Drvo kao građevni materijal budućnosti. Građevinar.

[4] Bogdan A. (2020). Drvo u modernome graditeljstvu. Građevinar.

[5] Drvo Kao Građevinski Materijal i Prirodna Sredstva za Njegovu Zaštitu|Terrabija. http://terrabija.com/2016/08/02/168/.

[6] Schädle P., Blaß H.J. Erthquake behaviour of modern timber construction systems. Proceedings of the 11th World Conference on Timber Engineering 2010 (WCTE 2010).

[7] Luxhome. https://www.luxhome.at/.

[8] Ecocell-Us-Building-System—Ecocell. https://ecocell.ch/en/us-building-system.

[9] Fabric Workshop—Mass Timber Solutions. https://www.fabricws.com/building-solution.

[10] Innovative ‘Wooden Bricks’ System Cuts Building Time to Just a Few Days ArchDaily. https://www.archdaily.com/887541/innovative-wooden-bricks-system-cuts-building-time-to-just-a-few-days.

[11] Insulated Formwork Block for Self-Build Project—Gablok. https://gablok.be/en/.

[12] STEKO^®^ Building Systems|Everybody Wins with Smart Construction. https://www.stekosouthamerica.com.

[13] Fonseca E.M.M., Gouveia P.J.V. (2018). Analysis of simply supported wood beams at ambient and high temperatures. Int. J. Eng. Technol..

[14] Wan Mohamad W., Razlan M.A., Ahmad Z. (2011). Bending strength properties of glued laminated timber from selected Malaysian hardwood timber. Int. J. Civ. Environ. Eng..

[15] Okamoto S., Akiyama N., Araki Y., Aoki K., Inayama M. (2021). Study on the strength of glued laminated timber beams with round holes: Difference in structural performance between homogeneous-grade and heterogeneous-grade timber. J. Wood Sci..

[16] Abaqus SIMULIA|Nonlinear Finite Element Analysis (FEA) Software. https://www.4realsim.com/abaqus/.

[17] Abaqus Material Data Definition. https://abaqus-docs.mit.edu/2017/English/SIMACAEMATRefMap/simamat-c-materialdata.htm.

[18] Jordaan J. (2018). Four-point bending fatigue test specimen design by FEA. R&D J..

[19] Khorsandnia N., Valipour H., Crews K. (2014). Structural response of timber-concrete composite beams predicted by finite element models and manual calculations. Adv. Struct. Eng..

[20] Eurocode 5: Design of Timber Structures—Part 1-1: General—Common Rules and Rules for Buildings (EN 1995-1-1:2004/A2:2014). https://www.phd.eng.br/wp-content/uploads/2015/12/en.1995.1.1.2004.pdf.

[21] Kawecki B., Podgórski J. (2020). 3D abaqus simulation of bent softwood elements. Arch. Civ. Eng..

[22] Herranen H., Pabut O., Eerme M., Majak J., Pohlak M., Kers J., Saarna M., Allikas G., Aruniit A. (2012). Design and testing of sandwich structures with different core materials. Medziagotyra.

[23] Souliman M.I. (2021). Preliminary Finite Element Modeling of Asphalt Material Using Ansys. Civ. Eng. Limits.

[24] Baño V., Arriaga F., Soilán A., Guaita M. (2011). Prediction of bending load capacity of timber beams using a finite element method simulation of knots and grain deviation. Biosyst. Eng..

[25] Zhang J., Xu Q., Xu Y., Zhang M. (2015). Research on residual bending capacities of used wood members based on the correlation between non-destructive testing results and the mechanical properties of wood. J. Zhejiang Univ. Sci. A.

[26] (2012). EN 408:2012: Timber Structures—Structural Timber and Glued Laminated Timber—Determination of Some Physical and Mechanical Properties. https://repozitorij.hzn.hr/norm/HRN+EN+408%3A2012.

[27] Ansys Inc. (2011). Ansys Meshing User’ s Guide. Renew. Sustain. Energy Rev..

